# A Review on *Alaria alata*, *Toxoplasma gondii* and *Sarcocystis* spp. in Mammalian Game Meat Consumed in Europe: Epidemiology, Risk Management and Future Directions

**DOI:** 10.3390/ani12030263

**Published:** 2022-01-21

**Authors:** Lisa Guardone, Andrea Armani, Francesca Mancianti, Ezio Ferroglio

**Affiliations:** 1 Department of Veterinary Sciences, University of Pisa, Viale delle Piagge 2, 56124 Pisa, PI, Italy; andrea.armani@unipi.it (A.A.); francesca.mancianti@unipi.it (F.M.); 2 Department of Veterinary Sciences, University of Turin, Largo Paolo Braccini 2, 10095 Grugliasco, TO, Italy; ezio.ferroglio@unito.it

**Keywords:** foodborne parasites, wildlife, hunting

## Abstract

**Simple Summary:**

In many European countries, game meat consumption is related to the traditional hunting culture. Its demand and consumption are increasing, also due to the growing populations of wild ungulates. However, specific public health issues exist and should be taken into account. This review focuses on the causal agents, epidemiology, potential risk for human health and its management along the supply chain, including parasite detection at slaughtering and inactivation in meat, of three parasites (*Alaria alata*, *Toxoplasma gondii* and *Sarcocystis* spp.), which can be transmitted by the main mammalian game meat species in the EU: wild boar (*Sus scrofa*), red deer (*Cervus elaphus*), roe deer (*Capreolus capreolus*), fallow deer (*Dama dama*), Alpine chamois (*Rupicapra rupicapra*), moose (*Alces alces*), hare (*Lepus europaeus*) and wild rabbit (*Oryctolagus cuniculus*). By presenting the main issues and knowledge gaps, this study aims to contribute to an improved control supporting the risk analysis process.

**Abstract:**

Game meat is increasingly appreciated and consumed in Europe, also due to the growing population of wild ungulates. In addition to interesting nutritional properties and market opportunities, game meat is characterized by some specific public health issues. This review focuses on the etiology, epidemiology, public health aspects and risk management along the supply chain, including parasite detection at slaughtering and inactivation in meat, of three selected foodborne parasitic hazards (*Alaria alata*, *Toxoplasma gondii* and *Sarcocystis* spp.) in the main mammalian game meat species in the EU: wild boar (*Sus scrofa*), red deer (*Cervus elaphus*), roe deer (*Capreolus capreolus*), fallow deer (*Dama dama*), Alpine chamois (*Rupicapra rupicapra*), moose (*Alces alces*), hare (*Lepus europaeus*) and wild rabbit (*Oryctolagus cuniculus*). The presented data point out the main issues, and knowledge gaps as well as the potential for improved control in order to contribute to the risk analysis process. To pursue an effective management of these parasitic zoonoses, awareness raising should involve all figures in the supply chain, including hunters, restaurateurs and consumers. Human behaviour and the lack of knowledge regarding meat borne parasitic zoonoses and the health risks they pose seem to be the most important factors responsible for human infections. However, detection methods, starting from the sampling procedure, should be further developed and standardized in order to improve the collection of accurate and up-to-date epidemiological data.

## 1. Introduction: Hunted Species, Hunting, Trade and Consumption Volumes in Europe

The term “game” covers a wide range of species, farmed, herded, kept in enclosures or caught in the wild, for which farming and hunting practices vary between European Member States [1]. The European regulation defines *“wild game”* as follows: “*wild ungulates and lagomorphs, as well as other land mammals that are hunted for human consumption and are considered to be wild under the applicable law in the Member State concerned, including mammals living in enclosed territory under conditions of freedom similar to those of wild game*’; and, ‘*wild birds that are hunted for human consumption.”* “*Farmed game*” refers instead to farmed ratites and farmed land mammals other than those reported above [2]. Even though European Union (EU) legislation does not provide a precise list of edible game species, an extensive survey on wild food in Europe identified 38 hunted species (26 birds and 12 mammals). The five main species, which are hunted in almost all EU countries and have the largest harvest numbers, are *Cervus elaphus* (red deer), *Capreolus capreolus* (roe deer), *Sus scrofa* (wild boar), *Lepus europaeus* (hare) and *Phasianus colchicus* (pheasant) [3]. Similarly, red deer, roe deer, wild boar and hare, with the addition of *Dama dama* (fallow deer) and *Oryctolagus cuniculus* (wild rabbit), were identified by Thomas et al. [4] as the most important game species hunted in the EU based on FAO data [5]. Other wild ungulates commonly hunted and consumed across Europe are Alpine chamois (*Rupicapra rupicapra*) [6] and moose (*Alces alces*) [7]. Interestingly, red deer, roe deer and wild boar are reported among the most important large mammal species hunted by Mesolithic people in Europe, as hunting for game meat is a human ancestral tradition [8]. However, the type and variety of consumed species strongly depends on the country [3].

The consumption of game meat is related to the traditional hunting culture, and this type of meat is commonly shared among hunters and their families [9,10]. However, in recent years, the popularity of hunted wild game meat increased in many developed countries, including Europe [11,12]. Currently, the game meat trade, within and among EU member states, as well as between Europe and other countries, is large and well established in some Member States and generates high revenues [1,3,5,11]. This is also connected with the increasing number of free-living wild ungulates, especially red deer, roe deer and wild boar, across Europe [13]. Data on volumes of hunted species were collected by FAO [5] by using a questionnaire. The 13 EU countries that provided the number of killed animals have around 5.5 million hunters, representing 82% of the almost 7 million hunters in the 28 European Member States in 2010.Assuming that a similar number of mammals are killed per hunter by the remaining 18%, an annual kill across the EU of over 6 million large game mammals (3 deer species and the wild boar), 12 million rabbits and hares and over 80 million birds can be estimated. This supports an international game meat market worth over 1.1 thousand million Euros [4].

Trade data were also collected [5], but fewer countries (n = 6) answered. Considering that the six EU countries that reported trade data have 26.56% of the total hunters reported in the EU in 2010, extrapolation of the ~300 million Euros reported by those countries provides an estimated export trade value of over EUR 1000 million a year for the entire EU. Although the relationship between the number of hunters and the level of trade may not be direct and, thus, the figure not exactly precise, it is still useful to have an idea of the large overall value of trade of the most important species, which were deer and wild boar, waterfowl, pheasant and other non-wetland gamebirds [4]. 

As regards estimations of annual game meat consumption in the EU, figures vary from 0.08 kg/capita in Poland and Portugal to 5.7 kg/capita in France. The fraction of the meat market covered by game meat also varies widely, accounting for 0.04% in Bulgaria to over 6.5% in France [3]. In Italy, wild game meat is estimated to represent only 0.1% of the apparent consumption of meat [14], although other estimates report high consumption values for Italian hunters’ families (up to 4 kg/capita per year) [3]. Higher consumption in hunters (up to 8.4 kg/capita per year) was also reported in Andalusia [3]. However, game meat is currently not only consumed by hunters and their families but may also enter the retail market and restaurants [3]. Consumption trends are strongly influenced by consumers’ attitude towards hunted game meat, but the existing literature on the topic focuses mainly on non-European countries, such as Africa and Australia [12]. However, the results from these international studies cannot be extended to other geographical contexts [15], as game meat consumption is strictly related to the availability of local species and to socio-cultural heritage. Only a few studies assessing European consumers’ attitudes and purchase behavior towards hunted game meat products are available [12]. The most exhaustive investigated consumers’ attitudes and perceived quality of game meat in ten countries (Czech Republic, Poland, Slovakia, Croatia, Albania, Bosnia and Herzegovina, Bulgaria, Former Yugoslav Republic of Macedonia, Montenegro and Serbia), showing that the consumption rate is influenced by a number of factors, such as location, age and gender, and highlighted, for example, that the consumption is higher in Southeast European countries, particularly among men and older consumers [15]. The remaining studies concern single Europe countries such as Sweden [7], Hungary [16], Poland [17,18] and Italy [6]. The growing trend of game meat consumption also responds to modern consumers’ concerns related to the sustainability of the meat supply chain [11,19], as these products present interesting environmental and nutritional characteristics. In fact, this meat is often perceived as more environmentally friendly than conventional meat [20] and presents excellent nutritional characteristics in terms of protein and fat contents [6]. On the contrary, strong aversion to hunting practices by another part of the society exists, mainly due to animal welfare issues [21].

As regards organoleptic properties, it is known that game meat has a specific taste. However, since it is mostly derived from free living animals, taste and quality may change consistently depending on several factors, such as the animal species, age, sex, health condition, habitat and diet as well as factors related to the death of the animal and postmortem treatments [22], which may also impact on carcass hygienic conditions [23,24]. Therefore, the full application of the current European legislation requirements to place wild game on the market could play an important role also in this regard [20].

In addition to opportunities and growing interest in the sector, safety issues exist and need to be addressed, as consumers’ concerns may also influence the level of consumption. Recently published reviews on public health issues associated to game meat, including microbiological [25,26,27], or heavy metal [4] contaminations are already available. As regards foodborne parasitic zoonoses, transmission can occur by the ingestion of meat contaminated by parasites developmental stages [28,29]. Among these, the relevance of *Trichinella* spp. for game meat, and particularly for wild boar, is well known [30,31,32,33]. The risk management of this parasite is regulated along the food chain by a specific legislation [34], as well as by up-to-date recommendations on diagnostic aspects [35,36] and pre-harvest and post-harvest control [37,38] issued by the International Commission on Trichinellosis. On the contrary, the availability of official documents for the management of other meat-borne parasitic hazards is lacking. Among these, *Toxoplasma gondii* has a well-known relevance in game meat safety [28,39,40,41], while *Alaria alata* and *Sarcocystis* spp. are considered emerging and neglected [29,42,43]. Therefore, this study aims to review the etiology, epidemiology, public health aspects and risk management along the supply chain, including parasite detection at slaughtering and inactivation in meat, of *T. gondii*, *Alaria alata* and *Sarcocystis* spp. in the main mammalian game meat species consumed in the EU (*S. scrofa, C. elaphus, C. capreolus, D. dama, R. rupicapra, A. alces, L. europaeus* and *O. cuniculus*), to contribute to the risk analysis process on these issues potentially affecting the growing sector of game meat. 

## 2. Materials and Methods

A literature search was conducted on PubMed, Google Scholar and Web of Science using “game OR wild OR venison” AND meat AND “consumption OR food safety OR public health OR zoonosis OR parasites” AND Europe (and different combinations of the same terms) as search strings. In addition, a second specific search was conducted using common and scientific names of mammalian species (wild boar—*S. scrofa;* red deer—*C. elaphus;* roe deer—*C. capreolus;* fallow deer—*D. dama;* Alpine chamois—*R. rupicapra;* moose—*Alces alces;* European hare—*L. europaeus;* wild rabbit—*O. cuniculus*) combined with the names of the selected parasites or related disease (*Toxoplasma* OR toxoplasmosis; *Alaria* OR alariosisi; *Sarcocystis* OR Sarcocystosis). No time limit was set. The selected articles included reviews, case reports, outbreak investigations, observational and surveillance surveys, while conference reports and dissertations were excluded. Additional literature was found using a snowball search. The search methodology met the recommended criteria for transparency and reproducibility of narrative reviews [44]. 

For each of the three selected foodborne parasitic hazards, data were structured in the following sections:

1. Etiology, distribution and life cycle
2. Epidemiology in mammalian game species in the European Union
3. Public health aspects
4. Risk management along the supply chain, including parasite detection at slaughtering, identification and parasite inactivation in meat

## 3. *Alaria alata*

### 3.1. Etiology, Distribution, and Life Cycle

*Alaria alata* is a trematode distributed in Europe and the former Soviet republics. Other *Alaria* species are found in North and South America. *Alaria* spp. have a complex life cycle with carnivores as definitive hosts and two intermediate hosts. Eggs, which are excreted with the definitive host’s feces, release the miracidium larva typically in an aquatic environment. Miracidia actively search for a suitable freshwater snail (such as *Planorbis* spp. and *Heliosoma* spp.), which is the first intermediate host. Subsequently, infective furcocercariae leave the snails searching for a second intermediate host, classically an amphibian, where the parasite develops into a larval stage called mesocercaria, which is also known as *Distomum musculorum suis* (DMS). This stage may be ingested either by a definitive host or by a broad spectrum of paratenic or transport hosts, including mammals, reptiles and birds, where the parasite survives without further development [9,45]. The mesocercaria (DMS) passes through the paratenic host intestinal wall and reaches several anatomical districts, thus favoring the persistence and expansion of the parasite life cycle, as it can be transmitted between paratenic hosts following predation or scavenging [29]. Consequently, infection rates may be particularly high in omnivores, such as wild boars, living in water-rich areas where all host species (snails, amphibians and definitive hosts) are present [45]. 

### 3.2. Epidemiology in Mammalian Game Species in the European Union

Considering the above-described life cycle, the only relevant species for alariosis among those taken into account in this review is the wild boar. Although epidemiological studies on *A. alata* in Europe had mainly focused on carnivore definitive hosts [46,47,48], the accidental finding of mesocercariae in wild boar meat during routine *Trichinella* inspection in Croatia and Germany in the early 2000 ([45] and references therein) prompted epidemiological investigations on these hosts in several European countries. These showed that the parasite is actually widely distributed [49], with variable prevalence rates (Table 1), also influenced by the detection method used (*Trichinella* Inspection Method—TIM; or *Alaria* mesocercariae Migration Technique—AMT (see Section 3.4)). 

These data confirm that differences may be attributed to environmental conditions, as regions rich in watery areas are an ideal habitat for *A. alata* intermediate hosts [54,58,61]. Furthermore, an influence of seasonality, with higher prevalence rates in spring/summer, was observed [49,50]. 

### 3.3. Public Health Aspects

*Alaria* spp. mesocercariae, or DMS, represent a potential source of human infection. Reports of human alariosis have been reported exclusively from North America and attributed to *A. americana* or other *Alaria* spp., following ingestion of undercooked paratenic hosts (wild frogs and goose) [62,63]. Mesocercariae were shown to be able to reach all abdominal organs, lungs, eye, somatic muscle and subcutaneous tissue, resulting in intraocular infection, dermal and pulmonary symptoms and even systemic dissemination with fatal outcome [64,65,66,67]. The lack of pathognomonic symptoms and atypical multi-organ changes, as well as the lack of eosinophilia (typical for other parasitic invasions) and of specific serological tests, makes the diagnosis of human alariosis difficult [54]. 

Human infection by *A. alata* has not been detected so far [62,63]; thus, its pathogenicity has not been studied [9]. However, the close relation of *A. alata* to *A. americana*, the apparent lack of definitive host specificity, the ability of *A. alata* to infect primates and the high prevalence rates in wild boar in some areas, suggest a real zoonotic foodborne potential in Europe and the need for preventive measures to exclude this parasite from entering the food chain [61,63]. 

### 3.4. Risk Management along the Supply Chain

Pre-harvest risk management, which would eventually be feasible only for farmed wild boar, is limited by the complex life cycle and the vast range of second intermediate and paratenic hosts. With respect to post-harvest risk management, there are currently no specific regulations for the detection of *A. alata* in meat. 

#### 3.4.1. Parasite Detection at Slaughtering and Identification

Examination with the naked eye does not allow detecting DMS in the host carcass; thus, appropriate laboratory diagnostics are needed [56]. Since there is no legislative obligation for searching *A. alata*, this trematode is mainly detected during official *Trichinella* inspections [9,45,54]. However, some aspects limit the sensitiveness of this technique in detecting *Alaria* spp., resulting in a possible underestimation of DMS presence [54]. Firstly, differences in biology and spread pathways of *Trichinella* and *A. alata* result in diverse predilection sites. In fact, mesocercariae in wild boar tissues are heterogeneously distributed, mainly in the omentum, larynx, peritoneum and diaphragm, suggesting a predilection for muscle tissue containing high amounts of adipose and connective tissue and/or glandular tissues [43]. Thus, for detecting *A. alata,* targeted sampling involves the diaphragm as well as the pharynx area, particularly muscle, connective, adipose, glandular and lymphatic tissues [54], while when testing for trichinosis, removing fat and connective tissue from the sample is advised [36]. Moreover, a higher susceptibility of DMS towards the digestion fluid was observed, inducing death or damage and resulting in the loss of their motility [45], which represents a major diagnostic feature [43]. In addition, the mesh size of the sieve used for *Trichinella* is inappropriate for *A. alata* mesocercariae, as these are larger and may be retained [43,60]. Therefore, the presence of *A. alata* might be underestimated if based on *Trichinella* inspection, and a more sensitive approach, the *Alaria* mesocercariae migration technique (AMT), was developed and validated [68]. Direct comparison of the two methods demonstrated that the sensitivity of AMT to detect DMS in tissues of wild boars is nearly 60% higher than that of TIM [68]. Hence, the AMT method was applied by a number of laboratories, including EU Reference Laboratories [61]. Studies using both techniques confirmed that AMT provides 5.0 (95% CI 3.6–6.8) times higher rates of finding a positive sample than TIM [49,60], even though a recent study also pointed out that in several samples *A. alata* mesocercariae were only found with TIM, or in greater numbers than with AMT, suggesting that DMS may not be equally distributed in muscles [49].

In the case of detection, the parasite identification has mainly relied on microscopic observation of morphological characters, detailed in Möhl et al. [45], while molecular identification has been scarcely applied. Available molecular data consist in a limited number of partial DNA sequences of internal transcribed spacer 2 (ITS2), 28S ribosomal RNA, and cytochrome C oxidase subunit I (COI) [54]. The molecular analyses of 18S rDNA and COI genes, conducted in a recent study in Poland, demonstrated genetic variability between different isolates at the COI gene, reporting intraspecific genetic differences in European *A. alata* isolates for the first time. Further investigations are needed to understand the extent and significance of such findings [54].

#### 3.4.2. Parasite Inactivation in Meat

Heat treatment is reported as the most effective method for the inactivation of *A. alata* mesocercariae in wild boar meat. Heating at 72 °C for 2 min is recommended by the German Federal Institute for Risk Assessment [9], and, in other studies, DMS did not survive heating temperatures over 60.0 °C nor exposure to microwave heating after 90 s of treatment [69]. In contrast, tolerance towards cold temperatures appears to be higher: mesocercariae survived refrigeration temperatures for 5 days in a French trial [70], up to 13 days in another study [69], and motile mesocercariae were recovered from chilled carcasses even 20 days post mortem [71]. An effective inactivation by cold is only guaranteed if the infected game meat is frozen to a core temperature of −13.7 °C for a minimum of 2 h [69]. 

Several studies investigated the resistance of *A. alata* mesocercarie to curing, fermentation, cold smoking and drying in raw-cured meat products and showed that infection through the consumption of such products can be largely ruled out if food technological procedures are carried out properly. However, a risk cannot be excluded in cases of very early consumption of these products. In fact, 11.9% of salami sausages and 18.2% of another type of raw sausage contained mesocercariae 24 h after preparation. Therefore, even tasting the meat during production may represent a risk for infection with *A. alata* [29]. Such a risk is also related to the consumption habits of a given area. In Serbia, for example, a long-time tradition of homemade pork sausage production, which are commonly tasted and eaten when freshly made soon after slaughter, was reported [53]. Similar habits occur in Italy, where, in November 2012, uncooked sausages made with meat from uninspected wild boar were consumed by 38 persons in a village in Lucca province (Tuscany region, Italy). Of them, 32 developed clinical signs and symptoms of trichinellosis, and *Trichinella britovi* larvae were detected in vacuum-packed sausages made with the same batch of sausages consumed raw [72]. Thus, the consumption of this type of product represents a risk for several foodborne parasitic zoonoses.

## 4. *Toxoplasma gondii*

### 4.1. Etiology, Distribution and Life Cycle

*Toxoplasma gondii* is a zoonotic apicomplexan protozoan infecting a wide range of hosts, including humans, worldwide. Although *T. gondii* is the only species of the genus, molecular genotyping identified different parasite strains [73]. Three genotypes (type I, II and III) are mainly reported in Europe and North America, and a fourth clonal genotype was observed in USA, while in South America the genetic structure is more heterogeneous, with many non-clonal atypical genotypes [74].

Domestic and wild felids are the only known definitive hosts, shedding oocysts in feces, while a broad spectrum of warm-blooded species may act as intermediate hosts, where the parasite can persist within tissue cysts for their entire lifetime [75]. The indirect life cycle, described in detail in [76], is complex. *T. gondii* has three infectious stages: tachyzoites in groups or clones, bradyzoites in tissue cysts and sporozoites in oocysts. Therefore, the parasite may be transmitted via several routes: ingestion of sporulated oocysts, which may contaminate water, soil and food; transplacental dissemination of tachyzoites from mother to fetus; and ingestion of viable cysts mainly found in muscular and neural tissues of intermediate hosts [76]. 

### 4.2. Epidemiology in Mammalian Game Species in the European Union

Several species of wildlife are known to be susceptible and often highly parasitized [77,78,79,80]. Epidemiological data for each game species included in the review are provided below. Serological and molecular studies showed a level of infection of hunted wildlife ranging from 2% to 68% [81]. Comparison of the studies should be performed carefully, considering different methodologies, lack of standardized research methods, different types, number and collection methods of tested samples and geographical and climatic differences [10]. Omnivores and carnivores generally present higher infection levels, probably reflecting their higher likelihood to consume tissues infected with *T. gondii,* rather than an herbivore ingesting *T. gondii* oocysts from the environment. This is especially true when there is only one species acting as definitive host and contributing to oocyst environmental dissemination [77]. Nevertheless, the infection is present and widespread in many wild ruminant species. In particular, a study observed a significantly higher prevalence of antibodies against *T. gondii* in adult wild ungulates compared to subadults and yearlings, suggesting a cumulative likelihood for exposure, lifelong persistence of antibodies and horizontal transmission as the main route of infection in these species [82]. An effect of the habitat can also be observed, as animals living closer to inhabited areas seem to be at higher risk [77,83].

#### 4.2.1. Wild Boar

Most studies on *T. gondii* in wildlife have been conducted on wild boar, especially in recent years, although data are still lacking for many countries [84,85]. Variable seroprevalence rates were reported for wild boars tested in Europe (8–38%) ([10] and [79] and references therein) and worldwide [86]. Two meta-analyses were also recently published [85,87]: the global pooled seroprevalence of *T. gondii* among wild boars from 1995 to 2017 was 23% (95% CI: 19–27%), with a higher seroprevalence in North America and Europe (32% and 26%, respectively). While pooled seroprevalence values in males and females were similar, a higher prevalence was observed in wild boars >12 months of age, in agreement with other studies [85]. The other review focused on the Nordic–Baltic region, finding an estimated pooled seroprevalence of 33% in wild boars (CI95%: 26–41%), higher than domestic pigs, sheep and cattle [87].

#### 4.2.2. Red Deer and Roe Deer

Fewer data are available for cervids. Variable prevalence rates have been reported worldwide (6.6–53.5%) [88]. A recent systematic review and meta-analysis assessed the seroprevalence of *T. gondii* in roe deer and red deer in Europe [84]. Pooled seroprevalence was estimated to be 29% (95% CI: 23–35%) in roe deer and 15% (95% CI: 10–20%) in red deer. The highest pooled seroprevalence was observed for roe deer in Western Europe (40%; 95% CI: 31–49%), while it was only 21% (95% CI: 14%–28%) in Southern Europe. The higher pooled seroprevalence in roe deer than in red deer may be attributed to different oocyst occurrences in the habitats of the two species, as roe deer is a ubiquitous and more synanthropic species, thus living in areas where cats are present [77,84]. Another recent systematic review and meta-analysis on global seroprevalence of *T. gondii* in 12 species of “deer” from 1978 to 2019 found lower values for red deer (19.09%) and higher for roe deer (27.44%) [89].

#### 4.2.3. Fallow Deer

The global pooled prevalence in fallow deer was estimated to be 15.52% (683 animals in four studies) [89]. As regards specifically Europe, seroprevalences ranging from 15.6% to ~30% were found in Spain [82,83,90,91]. Lower values were found in the Czech Republic [92], while four farmed fallow deer in Belgium were all negative [93]. A statistically lower prevalence in fallow deer compared to sheep (10% and 47%, respectively) living together was found in Poland, and it was attributed to different grazing habits [88]. 

#### 4.2.4. Alpine Chamois

An Italian study on the alpine chamois (*Rupicapra r. rupicapra*) found a low prevalence of *T. gondii* both with serological (3.2%) and molecular (2%) analyses, and infections were concentrated in individuals >1 year-old, suggesting that, in this area, the chamois is a minor source of human infection [78]. Another study on the Western Alps found all 22 chamois negative [77]. However, a seroprevalence of 16.8% in an area of the French Alps in which European wildcats, lynx and domestic cats are present was reported [94]. 

#### 4.2.5. Moose

The recently estimated global prevalence was 8.94% [89], while the pooled seroprevalence in the Nordic–Baltic region was 16% (CI 95%: 10–23%) [87]. In fact, variable seroprevalences were reported from North Europe: 9.6% in Finland [95], 12.6% in Norway [96], 20% in Sweden [97] and 23.97% in Estonia [98].

#### 4.2.6. Lagomorphs

Rabbits and hares are infected with *T. gondii* via the ingestion of water and plants contaminated with oocysts excreted by definitive hosts with which they share the same habitats, thus being useful indicators of the level of contamination of the environment [99]. In addition, they may significantly contribute to *T. gondii* epidemiology as a potential source for other animals, allowing the parasite to be transmitted from one intermediate host to another (e.g., prey to carnivore) without the need of a felid definitive host [100]. Seroprevalence rates in the European rabbit (*O. cuniculus*) worldwide ranged from 0.9% to 37.5%, with most studies showing levels between 10 and 15% [99]. As for the European brown hare (*L. europaeus*), seroprevalence rates ranged from 0% to 46% among different European countries, but the dynamics of antibodies against *T. gondii* in hares are not well understood [100]. Studies in Sweden and Finland found hares to be negative. This evidence, together with cases of fatal toxoplasmosis in the same areas, suggests that hares may not survive toxoplasmosis in those countries. On the contrary, higher seroprevalence levels have been observed in other European countries, including France, Austria, Czech Republic or Slovakia [99,100], indicating a wide fluctuation from different areas, as already suggested [101]. 

### 4.3. Public Health Aspects

*Toxoplasma gondii* is among the most common foodborne pathogens and the most prevalent infections in humans. Seroprevalence rates ranging from 30% to 50% are estimated worldwide [102,103], although values vary across countries and different socioeconomic strata [104]. The relevant burden posed by this zoonotic parasite is well recognized, as it ranked fourth in the FAO/WHO global multi-criteria-based ranking for risk management of food-borne parasites [28] and third by the WHO-Foodborne Disease Burden Epidemiology Reference Group [41]. It was estimated that foodborne toxoplasmosis was responsible for 10.3 million (95% UI 7.40–14.9 million) cases in 2010, and 825,000 (95% UI 561,000–1.26 million) DALYs [105]. While toxoplasmosis may have serious consequences in certain specific groups, such as immunocompromised people and pregnant women, in immunocompetent adults the infection is generally asymptomatic or characterized by mild symptoms [75]. However, outbreaks of acquired toxoplasmosis suggested that the general population may occasionally be at risk, also depending on the strain involved [106,107]. For instance, acquired toxoplasmic retinochoroiditis may develop in ~2% of immunocompetent individuals after infection with *T. gondii* in the United States [108]. 

Although *T. gondii* transmission patterns differ depending on the country and the socioeconomic status, in Europe the consumption of raw or undercooked meat (or meat-derived products) from infected animals is often considered the most important source of human infection [39,75,79,109,110]. Approximately half of the game meat produced in Europe was estimated to be seropositive for *T. gondii*, and, accordingly, the meat of large-game species was identified as an important source of zoonotic toxoplasmosis [39], including meat from farmed deer and farmed wild boar [40]. Wild animals in northern Italy were pointed out as a potential source of atypical, recombinant and virulent genotypes [74], and genotype II, the main cause of human infections [111], was found in red deer in Central Italy [80]. Moreover, in the USA, a strain of *T. gondii* most commonly found in North American wildlife was recently shown to be responsible for 12 atypical cases of toxoplasmosis [112]. Indeed, epidemiological studies and several outbreaks have identified consumption of raw or undercooked game as a potential source of acute toxoplasmosis [107,112,113,114,115,116] even in pregnant women [109,114]. Additionally, also handling of game carcasses was shown to be a possible source of acquired ocular toxoplasmosis in previously healthy hunters [115,117]. Accordingly, a high risk of *T. gondii* infection in hunters has been reported in a study in Slovakia [118], and this parasite was ranked as a high priority for meat inspection in farmed deer and farmed wild boar [40]. Moreover, domestic manipulation could be a source of infection [39,75], and meat should not be tasted during preparation or cooking [39,109].

### 4.4. Risk Management along the Supply Chain

Pre-harvest control options for *Toxoplasma* would require strict rodent control and certification that the farm and surroundings are free of oocyst-shedding cats [79]. Vaccination of farm animals against tissue cyst formation or of cats against oocyst shedding has also been proposed, but its application is limited [79]. Obviously, these measures are applicable only for farmed game. On the contrary, post-harvest prevention may be used for both farmed and wild game. However, despite the fact that the disease burden of toxoplasmosis is probably as high as salmonellosis and campylobacteriosis, *T. gondii* in food animals is not monitored at slaughter, but screening is only performed incidentally and is mostly related to research projects.

#### 4.4.1. Parasite Detection at Slaughtering and Identification

*T. gondii* cysts in muscle cannot be macroscopically identified during meat inspection. Therefore, different direct or indirect methods, including biological, molecular, histological or serological techniques, alone or in combination, are used [75], generally on blood/serum, spleen, brain or muscle (frequently diaphragm and tongue) samples [10]. The most commonly used detection methods for meat are mouse bioassay, followed by cat bioassay and molecular methods. The bioassays can detect viable and infective *T. gondii*, but, in addition to ethical restrictions for the use of experimental animals, they are expensive and take long period of time before a result can be obtained [119]. Tissues samples can be examined by using immunohistochemical staining or applying molecular techniques for the detection of parasite DNA [76], by polymerase chain reaction (PCR), quantitative real-time PCR (qPCR) and loop-mediated isothermal amplification (LAMP). Quantitative real-time PCR is the most sensitive diagnostic technique; however, due to extreme simplicity and speed, LAMP provides a valuable alternative for fast and effective diagnosis in contexts where economic resources are limited and no valid alternative are available (e.g., game meat screening in rural areas) [81]. For genotyping, the PCR-RFLP method, together with the microsatellite analysis, is considered particularly useful due to its simplicity and inexpensiveness [74].

Different serologic procedures, such as indirect hemagglutination assays, indirect fluorescent antibody assays (IFAT), direct agglutination tests, latex agglutination tests (LAT), ELISA and the immunosorbent agglutination assay test [75], have been developed, although some (LAT, IFAT and indirect hemagglutination) may underestimate the prevalence of *T. gondii* [120]. Indirect assays based on the detection of *T. gondii*-specific antibodies may be used on live animals or freshly hunted carcasses [78,80,82] but not on food products [119]. However, a seropositive animal does not compulsively harbor active tissue cysts with infective parasites [121]. Depending on the characteristics of the applied method (e.g., discrimination between viable and non-viable parasites), and its performance (i.e., sensitivity and specificity), the results obtained should be evaluated differently. Moreover, most methods are not suitable for routine testing [119].

Recent development in molecular genotyping methods also allowed identifying parasite strains with high resolution and investigating transmission patterns among hosts. However, current data in the literature are still limited and fragmented [73]. Molecular epidemiology and population genetic studies have revealed widespread and distinct distribution of different *T. gondii* genotypes globally [73,122]. 

#### 4.4.2. Parasite Inactivation in Meat

*T. gondii* tissue cysts in meat were proven susceptible to various physical procedures such as freezing, heat treatment, irradiation, high-pressure, acidity and enhancing solutions [79,123,124]. Among post-harvest but pre-kitchen procedures, freezing would be one of the most practical risk management option as it can inactivate *T. gondii* tissue cysts, although different timing and temperature are necessary for 100% parasite killing efficiency [124]. *T. gondii* in meat is completely inactivated by freezing between −7 and −13 °C for 2–4 days [123]. There is no evidence that there are strains of *T. gondii* with different freezing susceptibilities [125]. However, the loss of sensory quality may be an important factor in consumers’ attitudes towards meat freezing. 

Alternatively, the primary control factor for the prevention of *T. gondii* infection via meat consumption is adequate cooking [39]. According to Dubey et al. [126], tissue cysts generally became nonviable by heating to 61 °C or higher temperature for 3.6 min. Thus, cooking times will vary depending on the thickness and the type of meat [126]. However, cooking style has an influence on cooking temperatures and time, and parts of meat being grilled or barbecued may not reach sufficiently high temperatures to kill the parasite [79]. The US FDA recommends cooking meat to a temperature of 62.8 °C and allowing it rest for at least 3 min [127,128], while a higher temperature (71 °C) is recommended for categories at risk, such as pregnant women [129].

Salting, curing and smoking can reduce the viability of tissue cysts. Variability in household conditions prevents safety recommendations [75], while hams derived from experimentally infected pigs and cured according to Parma ham (“*Prosciutto di Parma*”) consortium guidelines were analysed for viable parasites, finding no positive ham by either bioassay or in vitro culture and real-time PCR [130]. Industrial food processing technologies such as high pressure or gamma irradiation might also be used to inactivate the parasite, which are reviewed in [124].

## 5. *Sarcocystis* spp.

### 5.1. Etiology, Distribution, and Life Cycle

The genus *Sarcocystis* comprises over 200 species of apicomplexan protozoans affecting the digestive tract and muscle of several animal species, with a worldwide distribution. The cycle typically occurs between predators and preys. Carnivores (or omnivores) act as definitive hosts and excrete sporocysts in their feces, which may contaminate food or water and may be ingested by intermediate hosts. In intermediate hosts (herbivorous or omnivorous), *Sarcocystis* species form cysts in the musculature (sarcocysts), containing hundred thousand bradyzoites. This latter form represent the terminal asexual stage, which is infective for definitive hosts [131,132]. The first experiments demonstrating the obligatory two-host life cycle of *Sarcocystis* spp. date back to the early 1970s. Such studies also revealed that, contrary to previous beliefs, intermediate hosts may harbor more than one species of *Sarcocystis* (reviewed in Gjerde, [133]). Outcomes of more recent molecular investigations also showed that several *Sarcocystis* species are not as intermediate host specific as previously believed, either because they never developed such specificity or because they have recently become adapted to infect close relatives of their principal hosts due to human interference with natural environments during the past few centuries [134,135].

### 5.2. Epidemiology in Mammalian Game Species in the European Union

During the last decade, increasing interest in *Sarcocystis* spp. in wildlife has resulted in the description and molecular characterization of several new *Sarcocystis* species in various intermediate hosts [42]. Speciation seems to be especially complex among wild ungulates [131]. Detailed epidemiological data for each of the species included in the review are given below. Despite abundant information about *Sarcocystis* infection in wild ungulates in Europe and North America, prevalence data and infection intensity are rarely comparable, mainly due to the different methodologies applied (see Section 5.4), the muscle tissue selected for the analysis, the age of the sampled animals and the tropism attributable to some *Sarcocystis* spp. [136].

#### 5.2.1. Wild Boar

Wild boars, as domestic pigs, act as intermediate hosts of two *Sarcocystis* species: *S. suihominis* and *S. miescheriana* (synonym: *S. suicanis*). Humans are the definitive hosts of the first one, while the latter uses various members of the family Canidae, such as dogs, jackals, raccoon dogs, red foxes and wolves [137,138]. A third species, *S. porcifelis,* presumed to infect pigs and cats, was reported from the former Soviet Union in the 1970s, but its presence in pigs was not unequivocally confirmed ([137] and references therein). Several studies were conducted to determine the occurrence of *S. miescheriana* and *S. suihominis* in domestic and wild pigs in different countries, firstly differentiating them on the basis of the cyst morphology and subsequently by using molecular methods. The most recent surveys, all applying molecular techniques, investigated wild boars from Latvia [139], Italy [137], Romania [140], Spain [141] and Portugal [142] (Table 2). Previous reports are reviewed in Dubey et al. [131].

The more frequent occurrence of *S. miescheriana* is probably linked to the abundance of suitable definitive hosts (various canids) and consumption of wild boars by these carnivores [137]. The low prevalence of *S. suihominis*, instead, is likely due to a low occurrence of this species in humans, in combination with a low degree of environmental contamination with human stools [137]. Accordingly, other reports of *S. suihominis* in intermediate hosts (wild boar or pigs) in Europe seem to be quite rare and dated [143,144], the last being described 15 years ago [145], while the parasite is common in geographical areas with high levels of environmental contamination with human feces [146].

#### 5.2.2. Red Deer

The *Sarcocystis* species were intensively studied in cervids [147], a taxonomic group in which this parasitic genus appears to be poorly host specific [148,149,150] and for which the definitive hosts and many epidemiological aspects are still unknown [151]. To date, at least 11 different *Sarcocystis* species, forming sarcocysts of five major morphological types, have been molecularly characterized in European red deers [135,148,152] (Table 2). Other not fully recognized/identified species were also described in older studies (reviewed in Gjerde et al. [135]). Mixed natural infections with two or more species were frequently detected [148,151]. Two species, *S. hjorti* and *S. ovalis*, seem to be common in both red deer and moose; the other two (*S. hardangeri* and *S. tarandi*) mainly occur in reindeer, while *S. linearis* was described in red deer, roe deer and moose [135,152,153,154].

*S. hjorti* was the most frequent species in Norwegian red deer [148], and it was also reported from the same host in Lithuania [155] and Spain [135], suggesting a widespread distribution in Europe [151]. In addition, in Canton Grisons, Switzerland, *S. hjorti* was the most frequently detected species in red deers’ carcasses showing a grey-greenish discoloration associated with eosinophilic fasciitis, and it had been reported in animals showing this pathology in this region before. However, other *Sarcocystis* species (*S. venatoria*/*S. iberica*, *S. pilosa*, *S. linearis*/*S. taeniata* and *S. ovalis*) were also detected in the same study in animals with grey-greenish carcass discoloration, suggesting that different species might be involved in the pathogenesis of eosinophilic fasciitis/myositis in red deer [151], as observed in Belgium for cattle [156]. Furthermore, three *Sarcocystis* species were found in animals with normal carcasses [151]. Data on the pathogenicity of *Sarcocystis* spp. employing cervids as intermediate hosts are limited [154], and future studies are needed to better investigate the pathological significance of the various species.

#### 5.2.3. Roe Deer

The roe deer is a known intermediate host for at least six *Sarcocystis* species, *S. capreolicanis, S. entzerothi, S. gracilis, S. linearis, S. oviformis* and *S. silva,* identified by means of both microscopical and molecular methods [134,154]. Those species have been found in in roe deer in five European countries, including Italy [134], Lithuania [154,157], Poland [158], Spain [149] and Norway [159], also with high prevalence rates (Table 2). Very high prevalence rates, reaching 100%, had previously been shown in this host in Italy ([150] and references therein). According to molecular surveys, *S. gracilis* and *S. silva* seem to be particularly common, as they were identified from almost all the above-mentioned countries (Table 2).

#### 5.2.4. Fallow Deer

Fewer studies focused on fallow deer [147,149,160]. Although *Sarcocystis* spp. have been identified in fallow deer in older studies, in Germany, Italy, Austria and Spain (detailed in de Las Cuevas [149] and Cabaj et al. [147]), the species were only morphologically described by and not identified by molecular methods. On the contrary, more recent studies applying DNA-based techniques found three identified species in this host: *S. morae, S. entzerothi* and *S. gracilis* (Table 2). *Sarcocystis gracilis* and *S. entzerothi* had previously been reported from the roe deer in Italy and in Lithuania, respectively [134,154]. In Poland, all tested young fallow deer presented *Sarcocystis* spp., suggesting that a single year on the pastures was sufficient to become infected [147].

#### 5.2.5. Alpine Chamois

Only two dated cases of *Sarcocystis* spp. in Alpine chamois are known, in which cysts were morphologically identified. Boch and Schneidawind [161] described three *Sarcocystis* types, including one identified as *S. tenella*, while Odening et al. [162] isolated two types of *Sarcocystis*, identified as *Sarcocystis* sp. and *S. cornagliai*. The only available molecular study was conducted on three individuals of the subspecies *R. rupicapra tantrica* (Tatra chamois), an endangered species living in Polish and Slovakian Tatra mountains. On the basis of light microscopy and sequencing of COI and 18S rRNA genes, all sarcocysts were identified as *S. tenella,* which is typically associated to sheep, with dogs as definitive hosts [163]. Interestingly, all eight sarcocysts were isolated solely from the diaphragm and *latissimus dorsi* muscle, while none were detected in the myocardium, tongue and oesophagus, differently from sheep [163].

#### 5.2.6. Moose

Seven molecularly characterized *Sarcocystis* species were described in moose in Europe [153] (Table 2). Five of them (*S. hjorti, S. scandinavica, S. silva, S. alces,* and *S. ovalis*) were identified in Norway [164]. Recent molecular analysis in Latvia and Lithuania revealed six *Sarcocystis* species [153], of which four (*S. alces, S. hjorti, S. silva and S. ovalis*) had already been reported from this host, while *S. linearis*, already found in roe deer and red deer [134,135], was confirmed in moose for the first time. In addition, an unknown *Sarcocystis* spp. was detected [153]. High prevalence rates (>80%) were detected both in the Baltic States [153] and in Norway [164]. *S. alces* was the predominant species in the diaphragm and the esophagus in Norwegian moose [164], while other species (*S. hjorti, S. linearis, S. silva, S. ovalis* and *Sarcocystis* sp.) detected in the Baltic States were relatively rare [153].

#### 5.2.7. Lagomorphs

Two species of *Sarcocystis*, both having cats as definitive hosts, are generally recognized in the rabbit: *S. cuniculi* in *Oryctolagus* spp. and *S. leporum* in *Sylvilagus* spp. (cottontail). Epidemiological data on *Sarcocysts* in wild lagomorphs in Europe are less abundant compared to other species, and the reports are quite dated [165]. A low prevalence (7.3%) was found in the European hares hunted in Lithuania, while *Sarcocystis* was not found in 15 hares nor in 555 rabbits examined by other studies in the same country [155]. Commonly reported infection sites are tongue, esophagus, diaphragm, thigh, loin and thoracic wall muscle, where heavy infections can be observed grossly as thin pale streaks parallel to the muscle fibers, while mild infections are usually grossly unapparent [165].

### 5.3. Public Health Aspects

Sarcocystosis is a neglected parasitic infection [42]. Of the over 200 species known, three (*Sarcocystis hominis*, *S. heydorni* and *S. suihominis*) are known to be zoonotic, with transmission occurring through ingestion of muscle sarcocysts. The first two species are acquired following consumption of raw or undercooked beef, while *S. suihominis* is acquired through undercooked contaminated pork. Such human infections are generally restricted to the gastrointestinal tract. They may cause nausea, stomach ache, vomiting and diarrhea as early as 6–12 h after consumption, but mostly they are asymptomatic [132]. Thus, among the *Sarcocysts* species found in the most important European mammalian game species, *S. suihominis* is the only one with a confirmed zoonotic potential. Although there are no recent reports of human intestinal infections with *S. suihominis* in Europe [166], wild boars may be infected with *S. suihominis* [137,141]; thus, zoonotic risk following consumption of raw or undercooked meat from wild boars should not be neglected [137].

In addition, people may act as dead-end (aberrant intermediate) hosts for non-human *Sarcocystis* spp. after accidentally ingesting sporocysts, resulting in extraintestinal sarcocystosis. However, the species capable of aberrant infections are still poorly understood [132], and it has been hypothesized that seven or more species may be involved, based on differences in sarcocyst wall morphology observed in human tissue specimens [166]. Clinical symptoms may range from asymptomatic muscle cysts to a severe, acute and eosinophilic myositis associated with systemic symptoms and peripheral eosinophilia [132]. Furthermore, a toxin isolated from *S. fayeri* appears responsible for food poisoning in people who have consumed raw horsemeat [167].

Wildlife may harbor yet undiscovered zoonotic species for which people might serve as definitive hosts (following ingestion of sarcocysts) or even as accidental intermediate hosts (following ingestion of sporocysts) [132,166]. Recent outbreaks of “food poisoning” following consumption of *Sarcocystis* infected venison (deer) were described in Japan [168,169], suggesting a potential public health significance of *Sarcocystis* in cervids, which is so far poorly known [147]. In the first case, a 67-year-old Japanese man had eaten raw venison 4 h prior to the beginning of the symptoms (abdominal pain, vomiting, diarrhea and fever), which recovered in 24 h. Many white cysts containing bradyzoites, subsequently identified as *S. truncata* on the basis of the 18S rRNA, were found in venison meat [168]. Another outbreak was described in 2019, when 30 people showed analogous digestive symptoms after deer consumption. While bacteria and viruses were not detected in food samples and patient feces, molecular tests revealed DNA of *Sarcocystis* spp. in both deer meat and patient feces. Three types of *Sarcocystis* cysts were found in consumed deer meat, presumptively identified as *S. japonica*, *S.* cf. *tarandi* and *S. pilosa* based on their molecular and morphological characteristics. All three *Sarcocystis* spp. showed a positive reaction of immunohistochemical staining towards the 15 kDa protein deemed responsible for the toxicity of *S. fayeri* [169], as already observed for *S. sybillensis* and *S. wapiti* isolated from venison in another Japanese work [170], cited in Ota et al. [168]. Thus, the pathogenesis of the symptoms is still unclear, and further studies will be necessary to reveal if the 15-dalton protein is responsible for the enterotoxicity induced by *Sarcocystis* detected in venison [168]. The recent increase of the sika deer (*Cervus nippon*) population and the growing popularity of wild game cuisine may favor a rise in food poisoning following ingestion of raw venison in Japan [168], and public health issues should be better investigated also in Europe. 

### 5.4. Risk Management along the Supply Chain

As anticipated for the above-mentioned parasites, pre-harvest control measures are not applicable to wild game, while in post-harvest, hunters may have an important role in the life cycle of *Sarcocystis* spp. In fact, upon handling hunted carcasses, potentially infected viscera and carcasses can be left behind promoting dissemination. So far, specifically searching for *Sarcocystis* is not compulsory by law.

#### 5.4.1. Parasite Detection at Slaughtering and Identification

*Sarcocystis* spp. form cysts in the musculature (sarcocysts), which, with a few exceptions, are microscopic. Thus, their detection in the muscle may be conducted by compression, histology or tryptic digestion [136]. Normally, during the routine game meat control by official veterinarians, which includes the visual inspection and artificial digestion method in order to detect *Trichinella* larvae, the presence of sarcocysts in the fresh meat remains unobserved [140]. However, meat inspection aimed at detecting this parasite could be costly and time consuming, as sarcocysts should be detected by microscopic or antibody methods. Luzón et al. [136] conducted an analysis of correlation and agreement between two techniques (compression and histology) and muscles in red deer, showing wide variations in the prevalence and intensity of *Sarcocystis* infection. Higher intensities were observed in the heart irrespective of the method employed, while compression was significantly less sensitive than histology, especially with diaphragm samples. However, different locations were also attributed to different species (e.g., *S. cervicanis* in heart and diaphragm and *S. hjorti* only in diaphragm), suggesting that both heart and diaphragm should be sampled [136]. In general, the most commonly targeted tissues are heart, diaphragm, esophagus and tongue [136,149,154]. The influence of the type of muscle tissue examined is still debated. Although in a study no statistically significant differences in infection prevalence were observed between muscle groups in several species of cervids, others found higher infection intensity in samples of esophagus and heart muscle rather than in the diaphragm ([147] and references therein). On the contrary, Coelho et al. [142] found that histological sections of the diaphragm contained more sarcocysts than sections from the esophagus and heart, while Poli et al. [171] found samples from the heart that were more intensively parasitized than samples from the tongue, esophagus and diaphragm muscle. Furthermore, in another study, the initial histological screening of sections of the diaphragm was significantly less sensitive than the molecular assay, which was preceded by mechanical homogenization of about 25 g of each diaphragm sample. In this case, the difference was attributed to the smaller volume of tissue examined by histology, at least when only a single muscle section from each animal is examined [137].

The specific identification of the organisms should follow the detection. This was initially based on morphological characters, including size, wall thickness and structure, observed by scanning and transmission electron microscopy. However, these features can vary with different stages of cyst development, location and also with the fixation methods used [142]. Moreover, *Sarcocystis* species affecting cervids have a highly similar cyst morphology by light and electron microscopy [151]. Thus, the need to use DNA markers to discriminate *Sarcocystis* parasites arose. PCR methods can be applied to cysts recovered after compression or digestion [172]. For about 20 years, sequences from the nuclear ribosomal DNA unit, particularly of the 18S rRNA gene, were mainly used. Subsequently, COI was also introduced [134]. Thus, both markers have been used for differentiating closely related *Sarcocystis* species and for characterizing interspecific and intraspecific phylogenetic relationships [154]. Partial COI sequences were shown to perform better than 18S rRNA gene sequences for distinguishing closely related species using ruminants as intermediate hosts, whereas many *Sarcocystis* spp. using birds or carnivores as intermediate hosts differ very little, or not at all, both at COI and the 18S rRNA gene. Hence, other markers, such as the ITS1 region and the 28S rRNA gene have been used, alone or in combination [137,151]. Cloning was recently proposed to detect mixed infections [151], while Next Generation Sequencing (NGS) has so far been used only for detecting *Sarcocysts* spp. in water samples [173].

#### 5.4.2. Parasite Inactivation in Meat

Avoiding eating raw or undercooked meat prevents enteric infection with any of the three known *Sarcocystis* species for which people serve as definitive hosts (Section 5.3). In particular, *Sarcocysts* in pork can be destroyed at 60 °C for 20 min, 70 °C for 15 min or 100 °C for 5 min, or by freezing it at −4 °C for 2 days or −20 °C for 24 h. This is in agreement with general CDC guidelines which recommend cooking all non-poultry meat to an internal temperature of 71 °C [123]. According to another study, conducted on cysts from sika deer in Japan, *Sarcocystis* cysts in muscle lose viability following rapid freezing at −20°C, heating at 70 °C for 1 min and being placed in 2.0% salt for 1 day [174].

Furthermore, in order to prevent food poisoning caused by venison, it is essential to understand how to inactivate the toxin. In *S. fayeri*, this can be achieved by freezing [175]. A study assessed the viability of *Sarcocystis* spp. and the activity of their diarrheal toxin (a 15-dalton protein) in deer meat following cold storage, freezing, pH change and curing. The results showed that the species lost viability by freezing at −20 °C, −30 °C and −80 °C for <1 h, heating at 70 °C for 1 min, alkaline treatment (pH 10.0) for 4 days and the addition of salt at 2.0% for <1 day. Immunoblot assays showed that the diarrheal toxin disappeared together with the loss of viability. However, the parasite survived cooling at 0 °C and 4 °C and acidification (pH 3.0 and 5.0) for more than 7 days with the diarrheal toxin intact [174].

## 6. State of the Art and Future Directions

### 6.1. State of the Art

Data reviewed herein confirm that *A. alata*, *T. gondii* and *Sarcocystis* spp. are widely present in the main mammalian game meat species in the EU. Accordingly, around half of the European game meat was estimated to be seropositive for *T. gondii*, and the meat of large game species was considered as an important source of human toxoplasmosis [39]. Considering the growing population of wild ungulates in the EU and the ongoing consumption trends, the relevance of these zoonotic diseases may further increase.

In fact, diverse public health issues arise from the presence of these parasites in game meat products. *T. gondii* represents a well-renowned hazard, mainly associated to problems in immunocompromised people or pregnant women, but outbreaks of acquired toxoplasmosis showed that a potential risk exists also for the general population [176] and may be influenced by the strain [106,107]. In the USA, for instance, atypical toxoplasmosis cases were recently associated to a strain of *T. gondii* generally identified in wildlife [112]. Atypical, recombinant and virulent genotypes in wild animals were also found in northern Italy [74] and in the USA [177]. On the contrary, *A. alata* has not been identified as responsible for human infections so far [62,63], and zoonotic cases were only described in North America and attributed to *A. americana.* However, the closeness of the two congeneric species, as well as a low definitive host specificity, the demonstrated ability of *A. alata* to infect primates and its high prevalence values in wild boars in some European countries require a better characterization of its pathogenicity [9,63]. As regards *Sarcocystis* spp., in addition to the potential of wild boar for transmitting *S. suihominis*, humans may also become dead-end hosts for non-human *Sarcocystis* spp. after the accidental ingestion of oocysts, developing tissue sarcocystosis [63]. Wildlife in particular may host still unknown zoonotic species for which people might act as definitive or as aberrant intermediate hosts [132,166]. Furthermore, the recent outbreaks of food intoxications after consumption of deer meat found to be infected with *S. truncata* in Japan [168,169], similar to those caused by toxins described in *S. fayeri* (typically associated to horse meat), suggest the need to further investigate also this aspect [147,168].

The present review also pointed out several issues that should be addressed for implementing the risk management related to the presence of the aforesaid parasites. Traditionally, control of zoonotic parasites in host animals and their meat has been addressed at some level within the food chain, including on-farm biosecurity and inspection at the slaughterhouse. In particular, those applicable at the pre-harvest (farm) and post-harvest (primary processing in the slaughterhouse) phases are well described in the scientific literature and in guidelines developed by OIE, FAO and WHO [178]. As regards game, pre-harvest control is only feasible for farmed animals, while in wild game it is necessarily focused on post-harvest phases, starting from postmortem inspection. In order to pursue an effective management of these parasitic zoonoses, several aspects should be addressed, which are listed in the last section.

### 6.2. Future Directions

#### 6.2.1. Parasite Detection

Detection methods should be further developed and standardized, also to facilitate comparison among studies. In fact, with the possible exception of some *Sarcocystis* species [136], the presence of the three parasites object of this review is not detectable macroscopically by visual inspection. For *Toxoplasma*, for instance, technical challenges such as a lack of general agreement among the tests to be used, and the lack of simple, sensitive detection methods was already reported [63,79]. In order to improve data collection and to better evaluate the disease burden of toxoplasmosis, the Biological Hazard Panel of the European Food Safety Authority recommended that monitoring programs should be initiated in the pre-harvest sector on sheep, goats, pigs and also game [39]. However, no such program has been implemented [10]. The development of detection methods should also pay particular attention to the sampling procedure. For instance, *A. alata* was shown to have a different distribution in muscles, connective and fat in comparison with *Trichinella* [43], while for *Sarcocystis* the influence of the type of examined muscle tissue on the method sensitivity is still object of debate ([147] and references therein).

#### 6.2.2. Identification and Characterization

Parasite molecular identification and characterization would also deserve more research attention. Progresses in the field appear to be particularly relevant for the study of *Sarcocystis* epidemiology and host specificity [134,135], while there is a need to improve knowledge on genotypes for *T. gondii*, as current data in the literature are still limited and fragmented [73]. Genetic variability of the COI gene among different *A. alata* isolates was observed, but its significance needs to be further investigated [54].

#### 6.2.3. Legislative Framework

The lack of a specific legislative framework at the EU level that clearly establishes detection procedures and measures to be implemented in the case of positivity affects standardization with respect to detection and identification methods. In fact, while veterinary inspections and sampling of muscle tissue for the search of *Trichinella* are required only for wild boars that are intended to be commercialized [2,34], regularly monitoring other parasites also in other game species of increasing popularity is recommended [54]. In fact, Directive 2003/99/EC [179] states the necessity of monitoring zoonotic agents in animals by each member states, and, according to Commission Implementing Regulation (EU) 2019/627 [180], carcasses infected with parasites have to be declared unfit for human consumption. Some countries already started addressing these emerging issues. *A. alata*, for instance, was classified as a zoonotic agent of risk group 2 by the Swiss Federal Office for the Environment and the Federal Office of Public Health [181]. Similarly, the German Federal Institute for Risk Assessment (BfR) pointed out that although data were insufficient, a human risk could not be excluded and recommended declaring carcasses of wild boars found to be infected by *A. alata* mesocercariae during official inspection for *Trichinella* spp. unfit for human consumption [43]. However, the destruction of *A. alata* infected wild boar carcasses in Europe could then represent an economic problem [63]. Methods for a reliable inactivation of the parasites have been proposed as a possible solution to the dichotomy between preventive consumers’ protection on the one hand and monetary interests on the other hand [29].

#### 6.2.4. Awareness Raising

All figures involved in the supply chain, including hunters, restaurateurs and consumers, should be involved to increase awareness and knowledge. Hunters have a relevant role according to the Regulation (EC) No 853/2004 [2], which in Annex III, Section IVm on wild game meat states that “*Persons who hunt wild game with a view to placing it on the market for human consumption must have sufficient knowledge of the pathology of wild game, and of the production and handling of wild game and wild game meat after hunting*” and subsequently details training subjects as well as handling recommendations for large and small game. In fact, in order to avoid the transmission and spread of parasitic infections, viscera and carcasses should be properly managed. In particular, general hygienic procedures should be followed while processing raw game meat [83]. Moreover, in the last phases of the supply chain, the role of restaurateurs and consumers’ education is also important, as, if not submitted to other procedures able to inactivate pathogens/toxins, game meat should be cooked thoroughly before human consumption [83]. In fact, heat treatment is a secure method to inactivate the parasite, but while it is utopian to enforce recommendations on meat cooking to restaurants, consumers can request well-cooked meat or choose a dish, such as a stew, that contains well-cooked meat.

#### 6.2.5. Medical Education

Education on such pathogens should also be addressed to physicians and, specifically for *T. gondii*, ophthalmologists [117,177]. In fact, in a large outbreak of trichinellosis involving several patients in France and Serbia, where the contaminated wild boar meat originated from, diagnosis was delayed in part because the parasitosis was not known by most physicians, which resulted in complications in the French cases, such as facial paralysis and pulmonary embolism. Health alerts and survey networks are indispensable at a European level for controlling the disease [182]. Analogously, another author pointed out that trichinellosis remains a common problem in Bulgaria, but patients are often treated with antibiotics for weeks during winter due to misdiagnosis with pneumonia or influenza [183]. Lack of awareness of these parasitic diseases, delayed diagnoses and inappropriate treatments may result in a severe disease course and complications, while the lack of information that the illness is meat-borne may favor new exposures from the infected products [63,183].

## 7. Conclusions

Human behaviour and the lack of awareness regarding meat-borne parasitic zoonoses and the health risks they pose seem to be the most important factors responsible for human infections. However, detection methods, starting from the sampling procedure, should be further developed and standardized in order to improve the collection of accurate and up-to-date epidemiological data. Moreover, all three targeted diseases can also affect domestic animals raised for food production, including pigs, cattle and small ruminants, particularly in extensive farming systems. All the above discussed aspects show that a strict interaction between the public health and the animal health sectors is highly needed, pursuing a One Health approach.

## Figures and Tables

**Table 1:** Epidemiological studies or case reports regarding *Alaria alata* in Europe. TIM: *Trichinella* Inspection Method; AMT: *Alaria* mesocercariae Migration Technique.

Reference	Country	Prevalence	Method
Portier et al. [50]	France	0.6	TIM
Gazzonis et al. [51]	Italy	1.0	AMT
Berger et al. [52]	Hungary	1.6	AMT
Gavrilović et al. [53]	Serbia	3	TIM
Bilska-Zając et al. [54]	Poland	4.2	AMT
Paulsen et al. [55]	Czech Republic	6.8	AMT
Paulsen et al. [56]	Austria	6.7	AMT
Riehn et al. [57]	Germany	11.5	AMT
Maleševic et al. [58]	Serbia	4.4–26.0 ^1^	AMT
Riehn et al. [59]	Bulgaria	2 wild boars	AMT
Ozoliņa et al. [49]	Latvia	43.9	AMT
Ozoliņa et al. [60]	Latvia	76.7	AMT
Strokowska et al. [61]	Poland	44.3	AMT
Kästner et al. [62]	Germany	28.3	AMT

^1^ The two prevalence values refer to 2014 and 2015 and the difference was attributed to a flooding disaster occurred in May 2014.

**Table 2:** Studies reporting *Sarcocystis* spp. identified to molecular level in wild boar, red deer, roe deer, fallow deer and moose in Europe. P: prevalence.

Host Species	Reference	Country	P (%) ^a^	*Sarcocystis* spp.
Wild boar	Prakas et al. [139]	Latvia	87.1	*S. miescheriana*
	Gazzonis et al. [137]	Italy	97 1	*S. miescheriana* *S. suihominis*
	Imre et al. [140]	Romania	60.4	*S. miescheriana*
	Calero Bernal et al., 2016 [141]	Spain	72.2	*S. miescheriana, S. suihominis*
	Coehlo et al. [142]	Portugal	73.8	*S. miescheriana*
Red deer	Gjerde et al. [135]	Spain	-	*S. hjorti, S. linearis, S. cervicanis, S. iberica, S. venatoria, S. morae*
	Dahlgren et al. [148]	Norway	-	*S. hjorti, S. hardangeri, S. ovalis, S. rangiferi, S. tarandi*
	Basso et al. [151]	Switzerland	-	*S. hjorti, S. venatoria/S. iberica, S. pilosa, S. linearis/S. taeniata, S. ovalis*
Roe deer	Gjerde et al. [134]	Italy	-	*S. gracilis, S. silva, S. capreolicanis, S. linearis*
	Rudaitytė-Lukošienė et al. [154]	Lithuania	95	*S.* *capreolicanis, S. entzerothi,* *S. gracilis, S. linearis, S. oviformis, S. silva*
		Spain	100	*S. gracilis, S. linearis, S. silva*
	Prakas et al. [157]	Lithuania	-	*S. entzerothi*
	Kolenda et al. [158]	Poland	-	*S. gracilis*, *S. silva* *S. oviformis*
	Gjerde [159]	Norway	-	*S. gracilis*, *S. silva, S. capreolicanis*
Fallow deer	de Las Cuevas et al. [149]	Spain	66.7	*S. morae*
	Cabaj et al. [147]	Poland	-	*S. morae, S. gracilis, Sarcocystis* sp.
	Rudaitytė-Lukošienė et al. [160]	Lithuania	81.3	*S. morae* *, S. entzerothi*
Moose	Dahlgren et al. [164]	Norway	82.3	*S. alces, S. ovalis, S. scandinavica, Sarcocystis* spp. (type 1 and 2)
	Prakas et al. [153]	Latvia and Lithuania	81.7	*S. alces, S. hjorti, S. linearis, S. silva, S. ovalis, Sarcocystis* sp.

^a^ Prevalence values should not be compared as they were obtained with different methodological approaches. Moreover, they often refer to an overall *Sarcocystis* spp. prevalence rate and not to the single species.

## Data Availability

Data sharing not applicable.
